# Berry Flesh and Skin Ripening Features in *Vitis vinifera* as Assessed by Transcriptional Profiling

**DOI:** 10.1371/journal.pone.0039547

**Published:** 2012-06-29

**Authors:** Diego Lijavetzky, Pablo Carbonell-Bejerano, Jérôme Grimplet, Gema Bravo, Pilar Flores, José Fenoll, Pilar Hellín, Juan Carlos Oliveros, José M. Martínez-Zapater

**Affiliations:** 1 Departamento de Genética Molecular de Plantas, Centro Nacional de Biotecnología (CNB), Consejo Superior de Investigaciones Científicas (CSIC), Madrid, Spain; 2 Instituto de Ciencias de la Vid y del Vino (ICVV), Consejo Superior de Investigaciones Científicas -Universidad de La Rioja-Gobierno de La Rioja, Logroño, La Rioja, Spain; 3 Área de Biotecnología, Estación Sericícola, Instituto Murciano de Investigación y Desarrollo Agrario y Alimentario (IMIDA), La Alberca, Murcia, Spain; Instituto de Biología Molecular y Celular de Plantas, Spain

## Abstract

**Background:**

Ripening of fleshy fruit is a complex developmental process involving the differentiation of tissues with separate functions. During grapevine berry ripening important processes contributing to table and wine grape quality take place, some of them flesh- or skin-specific. In this study, transcriptional profiles throughout flesh and skin ripening were followed during two different seasons in a table grape cultivar ‘Muscat Hamburg’ to determine tissue-specific as well as common developmental programs.

**Methodology/Principal Findings:**

Using an updated GrapeGen Affymetrix GeneChip® annotation based on grapevine 12×v1 gene predictions, 2188 differentially accumulated transcripts between flesh and skin and 2839 transcripts differentially accumulated throughout ripening in the same manner in both tissues were identified. Transcriptional profiles were dominated by changes at the beginning of veraison which affect both pericarp tissues, although frequently delayed or with lower intensity in the skin than in the flesh. Functional enrichment analysis identified the decay on biosynthetic processes, photosynthesis and transport as a major part of the program delayed in the skin. In addition, a higher number of functional categories, including several related to macromolecule transport and phenylpropanoid and lipid biosynthesis, were over-represented in transcripts accumulated to higher levels in the skin. Functional enrichment also indicated auxin, gibberellins and bHLH transcription factors to take part in the regulation of pre-veraison processes in the pericarp, whereas WRKY and C2H2 family transcription factors seems to more specifically participate in the regulation of skin and flesh ripening, respectively.

**Conclusions/Significance:**

A transcriptomic analysis indicates that a large part of the ripening program is shared by both pericarp tissues despite some components are delayed in the skin. In addition, important tissue differences are present from early stages prior to the ripening onset including tissue-specific regulators. Altogether, these findings provide key elements to understand berry ripening and its differential regulation in flesh and skin.

## Introduction

Grapevine berries like other fleshy fruits are complex organs, formed by diverse tissues that follow common and differential patterns of development. Three clear parts can be distinguished in grapes, the exocarp (skin), the mesocarp (flesh), and the seeds. The exocarp and the mesocarp together constitute the pericarp that develops and ripens, shifting from the status of herbivores repulsive to herbivores attractive, to favor animal dissemination of the seeds [1]–[3]. The onset of ripening (veraison) signals the beginning of significant changes which include accumulation of sugar, berry softening, synthesis of anthocyanins, catabolism of organic acids and flavor maturation [4]. Many of the relevant traits for table or wine grape quality seem to be specifically related to only one of both pericarp tissues [3], [5]. For instance, the berry skin is the site of synthesis for anthocyanin pigments during ripening and therefore mainly responsible for berry and wine color [6]. This trait, very important for berry dissemination by birds in nature, also constitutes one of the external traits influencing fresh grape consumers. Additionally, since the flesh is generally white in *Vitis vinifera* cultivars, skin color development and stability is a major requirement in the production of red wines [7]. The skin is also the interface with the external environment and protects the inner tissues from pathogen attack and abiotic stress agents, which requires specialized constitution of the cell walls and layers of protecting waxes [8], [9].

Taste-related traits like sugar and acid content rely mostly on the flesh [10], [11]. During ripening the berry works as a sink organ and most of the sugars are imported to the flesh from source organs [12]. The major forms of stored carbohydrates in grapes are glucose and fructose, mainly derived from imported sucrose [13]. On the other hand, malic and tartaric acid levels during berry development determine total acid content. Malate accumulates at very high levels in the flesh of unripe berries and its content is drastically reduced during ripening [13], [14]. In contrast tartrate level remains constant after veraison and is higher at ripeness [10], [15]. An appropriate combination of sugar and acid is very important for flavor in table grape and also for the production of quality wines [13], [16]. Furthermore, proanthocyanidins, influencing red wine flavor by conferring astringency, are accumulated in the berry skin and seeds [17]–[20].

Other traits are influenced by both skin and flesh properties. This is the case of berry crispness and firmness, a desirable agronomic trait in table grape that has been related to mesocarp cell turgor as well as skin thickness and strength [21]–[23]. Likewise, aroma is a complex trait in the berry influenced by the accumulation of volatile and conjugated metabolites, mainly belonging to the biochemical families of esters, alcohols, terpenes, norisoprenoids and sulfur compound [3], [24]. For instance, the typical Muscat flavor, appreciated in table grape and in some special wines, is related to the accumulation of different monoterpenes in flesh and skin [25].

Although, it is conceivable that flesh and skin development and ripening are co-regulated within the berry developmental program, their different functions indicate that they must also undergo specific programs. In fact, environmental factors such as climate change-related temperature increases can uncouple the processes of flesh and skin ripening [26]–[28]. The complexity of grapevine berry ripening has been analyzed at the transcriptional level in different cultivars using either microarrays [29]–[34] or RNA-seq [35] as well as the interactions between berry ripening and abiotic agents such as water deficit [36] or agronomic practices such as cluster thinning [37]. However, a comparison of differential expression profiles among the different parts of the berry has only been addressed by Grimplet *et al*. [38] who compared the expression between skin, flesh and seed at harvest time. The study focused on the differences in the response to water deficit rather than on the developmental patterns on each tissue.

In this study, flesh and skin ripening was analyzed and compared at the transcriptomic level in the table grape cultivar ‘Muscat Hamburg’. Berries of this cultivar show high phenotypic values for skin color and Muscat flavor [39]. To perform this work the Affymetrix GrapeGen GeneChip® [40] with an improved annotation, based on the v1 gene predictions from the 12× grapevine genome sequence assembly [41] and new functional categorization of the transcripts represented, were used. Categorization of transcripts to an accurate level allowed for carrying out functional enrichment analysis to search for the processes taking place during berry ripening, identifying flesh and skin ripening distinctive marks. Results from a hypothesis-free approach confirmed the reliability of the experiment, while a directed analysis allowed for a deeper characterization of the developmental events involved.

## Results and Discussion

### Sampling and Evaluation of Grape Berry Ripening

Analyses of gene expression under field conditions represents a challenge since variation in environmental conditions can influence gene expression and ultimately grape berry ripening [27], [33], [36], [38], [42], [43]. To partially avoid environmental effects a sampling strategy based on monitoring developmental stages instead of a simple chronological sampling was followed. In addition, individual plants grown under a parral trellis conduction and controlled fertirrigation were used as biological replicates. The same plants were sampled in two consecutive growing seasons (2005 and 2006).

The berry ripening process was followed during nine weeks in five developing stages from pre-veraison to commercially ripen for a table grape variety. Samples were characterized for developmental and ripening parameters (Tables 1, S1, and S2). The sample for the earliest stage corresponded to the pre-veraison stage (P) characterized by hard green berries of at least 15 mm diameter with a maximum acid content (32 g/L) and minimum SSC (4.6° Brix) (Tables 1 and S1). The veraison samples, corresponding to the 50% veraison (V1) and 100% veraison (V2) stages, were selected on the basis of berry color (Table S1) that was acquired concomitantly to anthocyanin and total phenolics increase (Table 1). Most berry softening and growth took place between the first two sampled stages (Table 1). After veraison berries were collected at two different ripening stages (R1 and R2) based on their density (see Material and Methods). In this way, berries with density ranges from 110 to 130 g NaCl/L, corresponding to 17.3° Brix, and berries with density range from 130 to 150 g NaCl/L, corresponding to 19.5° Brix (Table S1), were considered. The highest anthocyanin and phenolics accumulation was measured between V2 and R1 (Table 1). In contrast, SSC rise and acidity decay evolved more progressively throughout stages after a marked change between P and V1 (Table S1), in parallel to monosaccharide accumulation and organic acid content reduction (Table S2). For the characterization of berry ripening at the transcriptome level, RNA was separately obtained from flesh and skin in all five sampling stages. For each stage, six biological replicates corresponding to three independent plants over two growing seasons were taken into account.

**Table 1:** Physiological data at different stages of berry ripening.

Parameter (year)	P	V1	V2	R1	R2
Equatorial diameter (2005)	15.8±0.1	18.6±0.2	18.9±0.2	19.2±0.3	19.9±0.0
Equatorial diameter (2006)	15.4±0.1	17.0±0.2	17.1±0.2	18.6±0.5	19.7±0.2
Berry fresh weight (2005)	2.5±0.2	4.2±0.4	4.6±0.3	5.2±0.3	5.1±0.3
Berry fresh weight (2006)	2.1±0.2	3.6±0.3	3.8±0.2	4.8±0.4	4.9±0.2
Berry dry weight (2005)	7.1±0.0	16.2±0.4	17.6±0.2	18.8±0.3	21.4±0.2
Berry dry weight (2006)	6.5±0.0	15.4±0.1	16.4±0.3	17.8±0.0	21.8±0.2
Firmness (2005)	100	16.19	14.32	14.10	13.90
Firmness (2006)	100	15.99	14.82	14.44	14.00
Total phenolic (2005)	281±2	314±4	335±5	1063±9	1191±7
Total phenolic (2006)	384±4	376±6	378±4	1095±10	1123±8
Total anthocyanin (2005)	N/d	296±5	545±5	2042±10	2280±5
Total anthocyanin (2006)	N/d	175±3	487±6	1797±9	2568±7

Equatorial diameter [mm], berry fresh weight [g], berry dry weight [%], firmness [%], total phenolic [µg·g^−1^ dry weight], and total anthocyanin [µg·g^−1^ dry weight] in ‘Muscat Hamburg’ ripening berries during 2005 and 2006 seasons. P, pre-veraison; V1, 50% veraison; V2, 100% veraison; R1, 110–130 g NaCl L^−1^; R2, 130–150 g NaCl L^−1^. Data are mean ± SD (n = 3).

### Main Variation Components in the Ripening Berry Transcriptome

**Figure 1:**
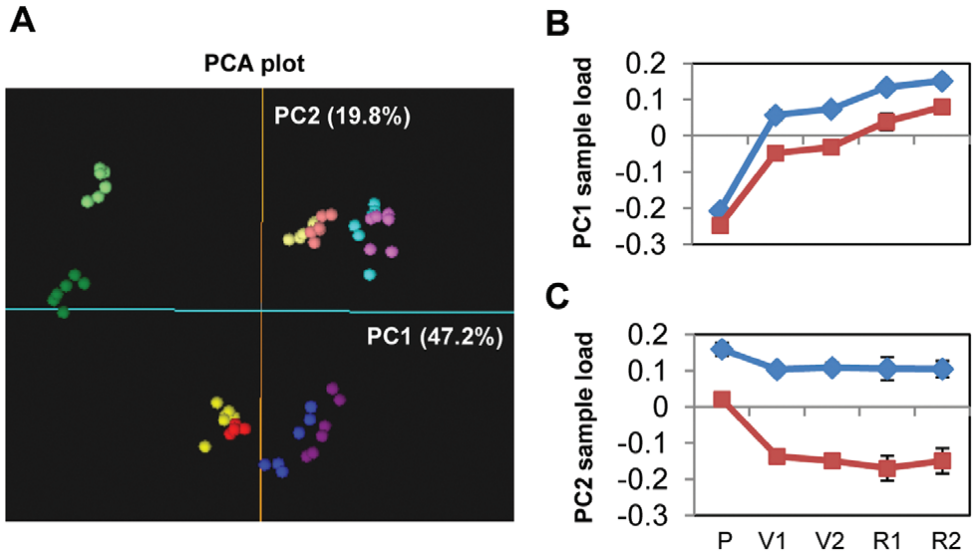
PCA plot of ‘Muscat Hamburg’ pericarp samples according to their expression data. **A**, PCA plot of flesh and skin ripening samples according to their RMA normalized expression data. The first (PC1) and the second (PC2) principal components are represented. Expression data of probe sets matching the same unique transcript were averaged before the PCA. Six samples corresponding to three biological replicas collected in two different years were analyzed per developmental stage. Green, pre-veraison (15 mm); yellow, 50% veraison; red, 100% veraison; blue, ripe 1; purple, ripe 2. Flesh: light colors; Skin: dark colors. **B**, Stage averaged PC1 loading scores for flesh and skin apart. **C**, Stage averaged PC2 loading scores for flesh and skin apart. At B and C, blue, flesh; dark red, skin. P, pre-veraison (>15 mm); V1, 50% veraison; V2, 100% veraison; R1, ripe 1; R2, ripe 2. Error lines indicate stage replicates standard deviation.

The above described five ripening stages were used for transcriptional profiling. As a first approach to analyze the complexity of the gene expression dataset, a principal component analysis (PCA) over the expression data of the 60 analyzed samples was performed. The first and second principal components (PC1 and PC2) explained together 67% of the variability in gene expression (47.2% and 19.8%, respectively) (Fig. 1). The results of the PCA plot showed consistency across biological replicas and growing seasons and, therefore, the experiment was considered highly reliable for further analysis. Only samples near the end of ripening appeared more variable and the variability was not only dependent on the year (data not shown). Experimental consistency allowed for extracting two main biological hints from the PCA results in a hypothesis-free approach: (i) PC1 corresponds to the ripening process itself and identified veraison as the stage when most prominent expression changes take place (Fig. 1). Both flesh and skin seemed to follow parallel ripening programs in the same direction. However, PC1 also showed delayed or less intense ripening-related activities in the skin than in the flesh at veraison, which seemed to be accelerated later. Recent experiments by Castellarin *et al*. [44] in the ‘Alicante Bouschet’ cultivar also indicate ripening to be initiated in the flesh. (ii) PC2 explained key differences between flesh and skin. Tissue differences were evident from pre-veraison and intensified during the following developmental stages, showing the largest differentiation at the beginning of veraison. No major differences in expression were found between 50% and 100% veraison in both tissues. PC3 explained 10.7% of the expression variability and could also be interpreted in biological terms by transient expression changes during veraison in both flesh and skin (Fig. S1; Table S3).

To further analyze the biological basis of the patterns observed in the PCA plot, transcripts that mostly contribute to each component were identified. This was done by considering the PCA loading scores (LS) for all the transcripts on each of the first three PCs (Table S3). The LS absolute value cut-off of ten was considered to identify transcripts dominating each PC (Fig. S2 A, C and D). In this manner, PC1 was dominated by transcripts up-regulated (PC1 LS >10) or, more frequently, down-regulated (PC1 LS <−10) during veraison and more slightly changing expression in the same direction afterwards. Broadly, the expression of those transcripts changed to a lesser extent and with some delay in the skin in comparison to the flesh (Fig. S2 B). PC2 was the result of tissue differential expression with transcripts displaying higher accumulation in the skin and with differences with respect to flesh being intensified during the ripening process (PC2 LS <−10). Few transcripts with higher expression in the flesh than in the skin (PC2 LS >10) also contributed to the distribution of the samples in the PC2 (Fig. S2 D). Finally, PC3 was based on transcripts that were specifically induced or repressed only during veraison in both tissues (Fig. S2 F).

Functional enrichment analysis according to the ‘GrapeGen custom functional annotation’ (Table S3) was carried out using FatiGO [45] to assess the biological significance of the transcripts showing a higher contribution to the expression variability. The lists of transcripts most contributing to each PC were compared to the rest of the transcripts represented in the GrapeGen GeneChip. Positively (PC LS >10) and negatively (PC LS <−10) highest scored transcripts were analyzed separately for each component. This functional analysis results indicated that the breakdown of several processes associated to photosynthesis was a major determinant biological process within PC1 (Table S4). Interestingly, ‘drought stress response’, ‘lipid transport’, ‘pectin modification’ and ‘beta-1,3 glucan catabolism’, related to cell wall metabolism, and ‘auxin-mediated signaling pathway’ were also over-represented among the PC1 higher negative scored genes. On the other hand, ‘xyloglucan modification’ and ‘oil body organization and biogenesis’ were significantly over-represented among the transcripts up-regulated after pre-veraison (PC1 LS >10).

Functional analysis of transcripts mostly contributing to PC2 revealed some processes more specifically related to skin ripening. ‘Macromolecule transport’ was the most significantly over-represented among the transcripts with higher expression in the skin (PC2 LS <−10) and more specifically its child category, ‘multidrug ABC transport’. Additionally, ‘secondary metabolism’ including ‘flavonoid biosynthesis’ together with ‘fatty acid metabolism’ were also significantly over-represented within this pattern. Part of these processes may be related to the phenolic ripening of the skin. No term was significant in the list of transcripts positively influencing the PC2 (PC2 LS >10, which corresponds to transcripts with higher expression in the flesh throughout the process).

### Transcriptional Bases for Flesh and Skin Differentiation

**Figure 2:**
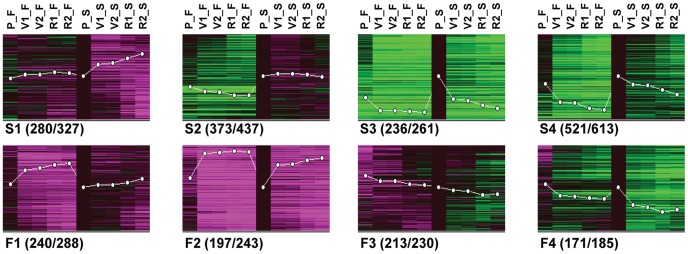
Expression patterns of transcripts differentially accumulated between flesh and skin throughout ‘Muscat Hamburg’ grape ripening. The 2584 significant probe sets (2232 unique transcripts) were allocated into 8 clusters. S. Profiles of transcripts with higher accumulation in the skin than in the flesh: S1, transcripts specifically or more highly up-regulated in the skin; S2, transcripts with constant expression in the skin and repressed in the flesh; S3, genes down-regulated in both skin and flesh; S4, transcripts either specifically, or more highly, or earlier down-regulated in the flesh. F. Profiles of transcripts with higher accumulation in the flesh than in the skin: F1, transcripts specifically or more highly up-regulated in the flesh; F2, transcripts up-regulated in both flesh and skin; F3, transcripts with a constant accumulation or slightly down-regulated in both tissues; F4, transcripts down-regulated in both flesh and skin. Expression data were normalized to pre-veraison skin: black, same expression; green, lower expression; magenta color, higher expression than in pre-veraison. White lines indicate the mean expression pattern of the clusters. Numbers in brackets correspond to non-redundant transcripts represented in the cluster/cluster included probe sets. P, pre-veraison (>15 mm); V1, 50% veraison; V2, 100% veraison; R1, ripe 1; R2, ripe 2.

Despite flesh and skin ripening processes are simultaneously activated, they generate tissues that are transcriptionally diverging over ripening as evidenced by the PCA analysis. Gene expression differences between both tissues were expressly analyzed to identify developmental programs that show tissues-specificities along ripening. A total of 2188 tissue differentially accumulated transcripts (Table S5) were identified according to a 1% FDR in a two-class time course significance analysis of microarrays (SAM) analysis with the fold-change cut-off described in Materials and Methods. These significant transcripts were grouped according to shared developmental expression patterns following a SOM (self-organizing map) analysis (Fig. 2). This clustering analysis indicated that the most recurrent expression differences between flesh and skin involved changes occurring in the same direction in both tissues but more intensely in the flesh. Concretely, the most prominent clusters (S3 and S4) contained transcripts down-regulated to a higher extent in the flesh than in the skin. This result is in agreement with the findings from PC1 on the biological basis of the ripening (see above). Apart from the delayed or attenuated ripening program in the skin, the berry skin also showed tissue-specific processes that became activated during ripening (cluster S1), which is in agreement with the findings for PC2. Another relevant group included transcripts with higher accumulation in the skin throughout all the ripening process, although without showing temporal expression changes (cluster S2). Transcripts more specifically up-regulated in the flesh were also identified (clusters F1 and F2).

Functional enrichment analyses were performed on each of these clusters to assess the biological significance underlying each tissue differential expression pattern. Clusters involving transcripts showing profiles of higher expression in the skin resulted in a higher number of significantly over-represented functional categories (clusters S1–4; Table S6) than their flesh counterparts. Therefore skin ripening could be considered in functional terms as a more diverse process than flesh ripening. Outstandingly, ‘phenylpropanoid metabolism’ was one of the skin tissue marks as assessed by functional enrichment in clusters S1 and S2. In the following paragraphs the developmental processes showing differential transcript accumulation profiles in flesh and skin that could be related to their specific developmental and ripening features will be highlighted.

#### Flavonoid metabolism gene expression is active in the skin before the onset of ripening

**Figure 3:**
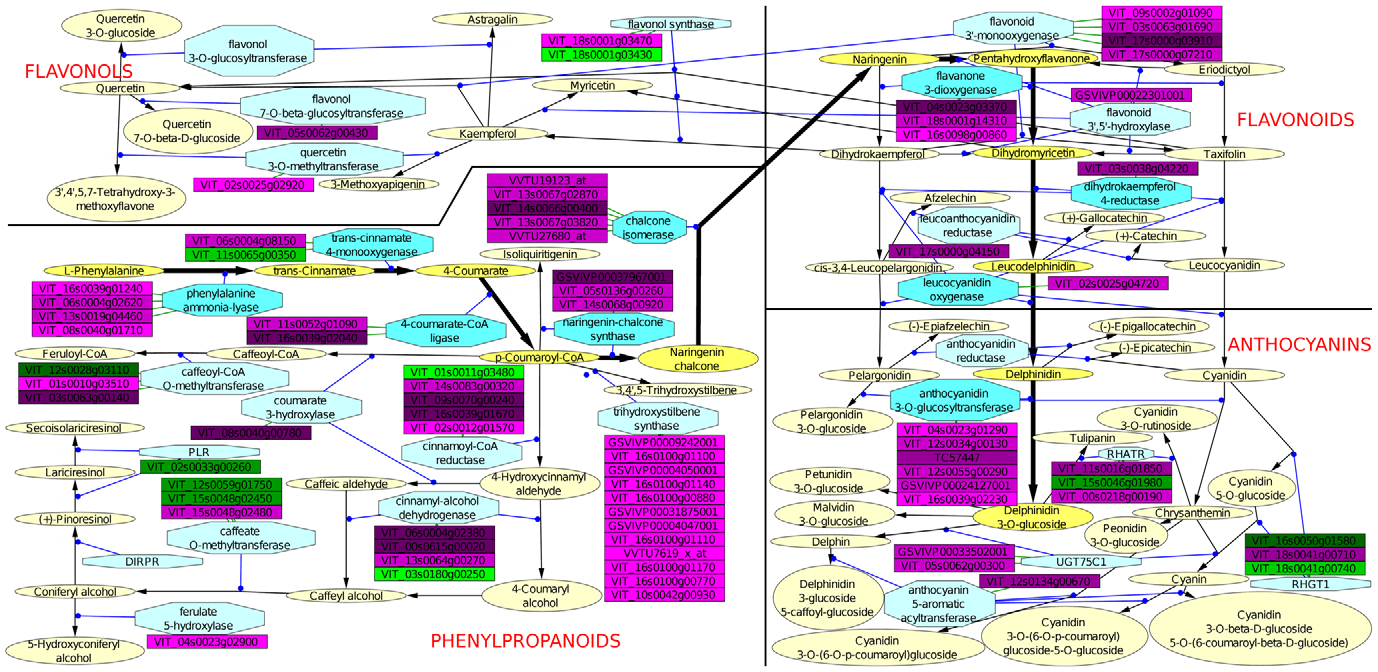
Phenylpropanoids-related transcripts showing significant differences between flesh and skin throughout ripening represented in VitisNet networks. Rectangles represent non-redundant transcripts differentially expressed; colors are defined according to the clusters represented in Figure 2: shades of green represent mRNAs with higher expression in flesh; shades of purple represent mRNAs with higher expression in skin. Lighter to darker colors represent cluster 1 to cluster 4.Yellow ovals: metabolites; blue hexagons: enzymes; black arrows: metabolic reactions; blue arrows: catalytic reactions; green arrows: translation reactions. Darker yellow and blue color shades and bold arrows highlight the reactions starting from phenylalanine leading to anthocyanidin biosynthesis.

Increased transcript expression concerning phenylpropanoid metabolism in the berry skin was one of the processes that most definitely differentiated both pericarp tissues (Tables S4 and S6). This is in agreement with the preferential accumulation for this kind of secondary metabolites in the external cell layers of the grapevine berry [6], [13]. Interestingly a branch of phenylpropanoid pathway, involving genes coding for enzymes in the flavonoid biosynthesis leading to anthocyanin accumulation, showed transcripts whose higher expression was established before the onset of ripening and maintained throughout the process (cluster S2 from Tables S5, S6, and Fig. 2). The phenylpropanoid pathway tissue differentially accumulated transcripts are mapped in the Figure 3, which included steady higher expression in the skin of two transcripts coding for chalcone synthase, four chalcone isomerase, one for flavonoid 3′,5′-hydroxylase, flavonoid 3′-hydroxylase, flavonoid 3-monooxygenase, leucoanthocyanidin dioxygenase, and several putative anthocyanidin glucosyl transferases. Moreover several transcripts coding for enzymes at the base of the phenylpropanoid biosynthesis pathway also showed constitutively higher expression in the exocarp, like two for phenylalanine ammonia-lyase, a 4-coumarate-CoA ligase, and a trans-cinnamate 4-monooxygenase. Strikingly, no conspicuous up-regulation of anthocyanin biosynthetic transcripts as a group related to the increase in anthocyanin biosynthesis upon veraison was found, when color acquisition takes place in ‘Muscat Hamburg’ berry skins (Table 1). However, the *VvGT1* transcript (*VIT_16s0039g02230*) coding for a UDP-glucose:flavonoid 3-O-glucosyltransferase (UFGT, [46]) was tremendously up-regulated between P and V1 in the skin and only modestly up-regulated in the pulp (cluster S1 in Fig. 3 and Table S5). UFGT is considered to catalyze the limiting step for anthocyanin accumulation [47], [48] and, indeed, the expression data support its role as a target for the activation of the process from the onset of ripening. It has been reported that *VvGT1* activation upon veraison is dependent on VvMybA1 TF [48], [49] and, in fact, this TF (*VIT_02s0033g00410*) also showed a prominent and stable up-regulation from veraison, being even more skin-specific than *VvGT1* (cluster S1 in Fig. 2 and Table S5). Thus, the transcriptomic data suggest that VvMybA1 directed *VvGT1* induction as a main mechanism triggering anthocyanin accumulation in the skin, as described for other cultivars. However, the existence of tissue-specific additional regulation of anthocyanin levels independent of transcription, such as increased anthocyanin accumulation during ripening due to changes in its degradation and/or rate of transport to the vacuole, cannot be ruled out.

#### Fatty acids demand appears more intense in the skin

Fatty acid metabolism showed transcriptional differences between flesh and skin (Tables S4 and S6). Transcripts coding for enzymes involved in most fatty acid anabolism steps were constitutively more expressed in the skin than in the flesh, from pre-veraison to ripeness (cluster S2 from Fig. 2, Table S5). It included transcripts for acetyl-CoA carboxylase (*VIT_18s0001g04980*), acyl carrier protein (*VIT_01s0244g00030*), beta-ketoacyl acyl carrier protein synthase condensing enzyme (*VIT_11s0016g00600*), fatty acid elongase (*VIT_15s0048g02720*), and fatty acid unsaturating enoyl reductase (*VIT_13s0019g01260*). Fatty acids might be metabolized in the exocarp for the biosynthesis of waxes during the biogenesis of the external cuticle layer. In fact, functional enrichment also reflected that wax biosynthesis was more active in the skin (Table S6) since three wax synthase coding transcripts (*VIT_15s0046g00710*, *VIT_15s0046g00660*, *VIT_15s0046g00520*) showed higher skin expression in all stages despite being progressively down-regulated from veraison (cluster S3 from Fig. 2 and Table S5). This expression trend is in agreement with the fact that exocarp cuticle thickness reaches its maximum around veraison [2], [50].

#### Macromolecule transport related gene expression remains more active in the skin throughout ripening

Several functional categories related to ‘macromolecule transport’ were significantly over-represented within different expression profiles involving higher gene expression in the skin. In this way, ‘multidrug ABC transport’ was significantly over-represented within transcripts showing steady higher accumulation in the skin (PC2 LS <10 and cluster S2; Tables S4 and S6). ATP-binding cassette (ABC) transporters compose one of the largest plant protein superfamilies; they participate in the active translocation of very diverse molecules across cell membranes [51] and, therefore, it is difficult to speculate on a specific biological function when being regulated as a group. Strikingly, ‘flavonoid biosynthesis’ and ‘anthocyanin-glycoside’ were also over-represented within cluster S2, whereas ABC transporters are reported to be involved in the vacuolar sequestration of such glycosides, in addition to glucuronides and glutathione conjugates [52], collectively being suggestive of the participation of some of the ABC transporters with higher expression in the skin in flavonoid conjugates accumulation there.

‘Lipid transport’ and ‘multidrug ABC transport’ were also significantly co-over-represented within transcripts more intensely down-regulated in the flesh than in the skin (PC1 loading score <10 and cluster S4, respectively; Tables S4 and S6). In such case, some of the transporters showing this expression pattern could be involved in the establishment of the external skin cuticle, considering that ABC transporters have been found essential for cuticle biogenesis in *Arabidopsis thaliana* epidermal cells [53]–[55]. In addition, ‘wax biosynthesis’ was also over-represented within transcripts repressed to a higher extent in the flesh as stated above. In fact, the over-representation of ‘lipid transport’ within highest PC1 negatively scored transcripts (i.e.: repressed in both tissues but to a higher extent in the flesh) was participated by the presence of several lipid-transfer protein encoding genes (Table S3); this type of protein has also been involved in plant cuticle assembly [56]–[58].

#### Accelerated breakdown of photosynthetic machinery gene expression correlates with an earlier activation of oxidative stress responses in the flesh

Photosynthetic machinery breakdown appears as a process dominating the transcriptional changes along the pericarp during ripening as indicated the hypothesis free approach (PC1 from the PCA, Table S4). This strongly suggests that photosynthesis is characteristic of pre-veraison berries and is inactivated during ripening, which involves a down-regulation in a vast number of genes given the complexity of the process itself. These expression profiles are in agreement with the gradual decline of photosynthetic activity initiated around the ripening onset observed in several varieties [59], [60]. Previous transcriptomic studies on berry ripening displayed similar decay in the photosynthesis pathway [30], [31] and such decay has also been shown at the proteome level throughout ‘Muscat Hamburg’ mesocarp ripening [61]. However, in the comparative flesh versus skin analysis, gene expression differences showed that this breakdown does not act equally in both pericarp tissues. Transcripts related to most photosynthesis processes, including dark and light reactions and pigment biosynthesis, were down-regulated stronger and earlier (from veraison instead of near maturity) in the flesh than in the skin (cluster S4 in Table S6). These results are in agreement with previous data from Grimplet *et* *al*. [38] showing that, in ripe berries, skins maintained greater expression of photosynthesis-related transcripts than the flesh.

Interestingly, the accelerated decay of photosynthesis in the flesh correlated with an earlier activation of oxidative stress-related genes (Cluster F2 from Fig. 2 and Table S6). Oxidative stress response genes activated to a higher degree in the flesh included, among others, a catalase encoding gene (*VIT_18s0122g01320*), two glutharedoxin encoding genes (*VIT_10s0003g00390*, *VIT_13s0067g01650*) as well as several genes involved in the metabolism of glutathione (*VIT_09s0002g06420*, *VIT_18s0089g00410*, *VIT_19s0093g00160*, and unmatched probe set *VVTU29518_x_at*) (Table S5). Activation of antioxidant systems such as the glutathione-dependent one in the flesh could be related to the rapid photosynthesis decay (cluster S4 in Fig. 2 and Table S6) and the reduced level of phenolic compounds [6], [13]. Activation of a comparable transcriptional response to oxidative stress has been previously reported in ‘Pinot Noir’ whole ripening berries around veraison, together with the occurrence of an oxidative burst rendering H_2_O_2_ accumulation [30]. Increased abscisic acid (ABA) [62], [63] and sugar accumulation in veraison berries (Table S2) might contribute to photosynthesis imbalance and thus increased reactive oxygen species production in the pericarp. Indeed, high sugar levels might result in a negative feedback on photosynthetic gene expression partially via ABA signaling such as in Arabidopsis [64] since it has been observed that ABA treatment of ‘Cabernet Sauvignon’ berry skins also causes a repression of photosynthesis-related and chlorophyll biosynthetic genes, correlating with induction of oxidative stress response genes [65]. On the other hand, the redox state of the chloroplast can also regulate expression of photosynthesis-related genes [66]–[68]. Thus, photosynthesis may become imbalanced earlier in the inner berry flesh with higher sugar content, promoting an earlier flesh oxidative burst and gene expression responses. A more exact determination of the origin and location of the oxidative burst in the berry might be relevant for understanding the mechanisms triggering the onset of grape ripening.

#### Oil body organization and biogenesis related gene expression appeared activated specifically during flesh ripening

‘Oil body organization and biogenesis’ and ‘C2H2 family transcription factor’ were the only categories significantly over-represented in the transcripts more specifically up-regulated in the flesh (cluster F1). There was no category enriched on the profiles including transcripts down-regulated during ripening and with higher expression in the flesh (cluster F3 and F4). However, the combination of these two clusters allowed for detecting enrichment of ‘cell wall metabolism’ and related categories, as well as of ‘auxin signaling’ (cluster F3+4, Table S6). Regarding oil-body-membrane protein genes enriched among those more specifically induced in the flesh (cluster F1 from Fig. 2 and Table S6), it resulted from the up-regulation after pre-veraison of a caleosin coding transcript (*VIT_05s0049g01780*) and three oleosin ones (**Table S5**; *VIT_14s0066g00700*, *VIT_02s0234g00110*, and one unmapped probe set *VVTU29901_s_at*). This type of proteins is specific of seed oil bodies, being important to the proper biogenesis and stability of this organelle [69]. However seed contamination was not expected within the flesh samples as supported by the absence of seed storage protein genes or other type of seed-specific process-related genes within the same expression pattern. To our knowledge no development of oil bodies related to grape flesh ripening has been reported; therefore, oil-body-membrane proteins may participate in novel uncharacterized functions for such case. For instance the induction of oil-body-membrane protein genes coincides with the post-veraison increased vacuolization of the cytoplasm in mesocarp cells [70], and they might be involved in the stabilization of tonoplast throughout ripening.

### Flesh and Skin Ripening Share Common Transcriptional Features

Although we have shown flesh and skin transcriptional ripening programs to differ significantly, PC1 and PC3 in the PCA plot evidenced both programs also sharing many common features, as expected for processes that are concomitantly activated (Fig. 1 and Fig. S1). To discriminate from tissue-specific processes those acting similarly during pericarp ripening, genes showing expression differences among the five developmental stages collected have been searched for after considering flesh and skin as the same sample type. In doing so, 2839 transcripts differentially accumulated during pericarp ripening were identified (Table S7), according to a Bonferroni corrected *p*-value <0.01 and a fold change cut-off described in Materials and Methods.

**Figure 4:**
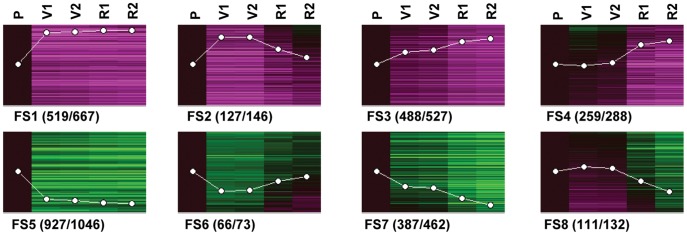
Expression patterns of transcripts with significant changes during berry ripening, and without significant tissue differences. The 3341 probe sets (2884 unique transcripts) were allocated into 8 clusters by SOM analysis. FS1, transcripts up-regulated after pre-veraison; FS2, transcripts up-regulated transiently at veraison; FS3, transcripts up-regulated progressively during ripening; FS4, transcripts up-regulated after veraison; FS5, transcript down-regulated after pre-veraison; FS6, transcripts down-regulated transiently at veraison; FS7, transcripts down-regulated progressively; FS8, transcripts down-regulated after veraison. Pericarp averaged expression data for each probe set are represented after being normalized to Pre-veraison: black, same expression; green, lower expression; magenta, higher expression. White lines indicate the mean expression pattern of the clusters. Numbers in brackets correspond to non-redundant transcripts represented in the cluster/cluster included probe sets. P, pre-veraison (>15 mm); V1, 50% veraison; V2, 100% veraison; R1, ripe 1; R2, ripe 2.

Differentially-accumulated transcripts were clustered within eight temporal expression patterns (Fig. 4) and, in agreement with the PCA analysis (Fig. 1), clustering showed that the highest number and magnitude of changes took place from P to V1 stages. Most of these changes involved activation (cluster FS1) or repression (cluster FS5) at veraison of genes whose expression was maintained afterwards. A smaller proportion of veraison changes took place transiently (cluster FS2, transiently up-regulated transcripts; cluster FS6, transiently down-regulated transcripts). Another considerable amount of transcripts changed expression progressively throughout ripening (cluster FS3, progressively up-regulated transcripts; cluster FS7, progressively down-regulated transcripts). Finally, transcripts up-regulated (cluster FS4) or down-regulated (cluster FS8) after the completion of veraison were detected. As observed with PCA, clustering showed minor differences between R1 and R2 sampling stages, whereas V1 and V2 samples were almost indistinguishable.

**Figure 5:**
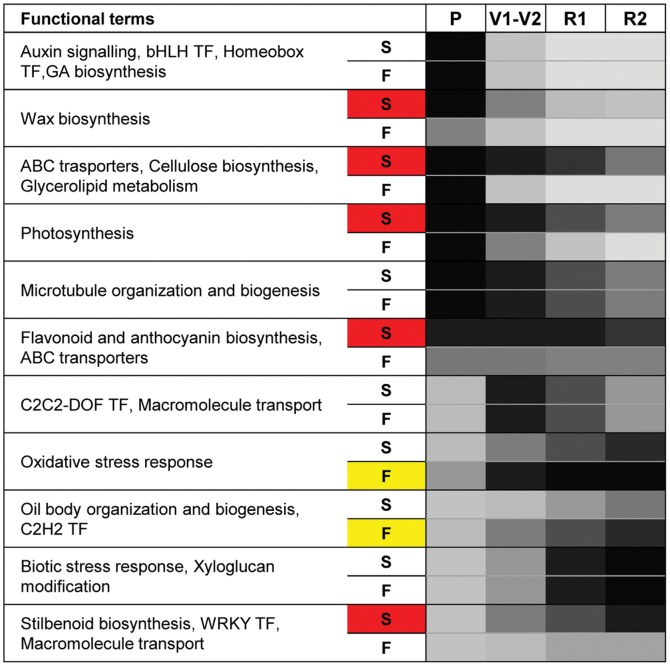
Pictogram summarizing the expression trend of enriched functional categories. Darker color indicates higher expression. S, skin; F, flesh. S and F are highlighted in red and in yellow, respectively in case of greater expression in the corresponding pericarp tissue. P, pre-veraison; V1–V2, 50 and 100% veraison; R1, ripe 1; R2, ripe 2.

Functional enrichment analysis was conducted over transcripts sharing the same developmental expression changes in flesh and skin. ‘Biotic stress response’ and ‘xyloglucan modification’ categories were enriched among transcripts induced around ripeness (cluster FS4). The developmental program switch at veraison was supported by the enrichment of ‘signaling’ and several child functional categories in cluster FS5 of transcripts down-regulated after veraison, indicating important regulatory changes. Those included ‘protein kinase’, ‘auxin signaling’ and ‘transcription factor’ involving bHLH (basic helix-loop-helix) and Homeobox domain families. ‘Metabolism’ and different child functional categories were enriched among progressively down-regulated transcripts (cluster FS7). This enriched category included ‘photosynthesis’, indicating a subset of genes related to that process commonly inactivated in both tissues in addition to the previously cited set of genes more intensely repressed in the flesh (see Figure 5 for a summary). It is worth to note that the ‘aquaporins’ functional category was enriched among the group integrating progressively and lately repressed transcripts (cluster FS7+8). Hence aquaporins repression after veraison appears as a shared feature to skin and flesh ripening. Berry water proportion also decreased throughout ripening, although mostly after the inception of veraison (Table 1). The aquaporin expression decay coincides with the cease of berry growth (Table 1); therefore, these proteins may play a role in the expansion of both pericarp tissues as proposed for ‘Cabernet Sauvignon’ [71]. In the following paragraphs, developmental processes taking place in parallel in flesh and skin, which can be detected after functional enrichment analysis of transcripts significantly changing accumulation throughout ripening, are discussed.

#### Cell wall biosynthesis gene expression is repressed during pericarp ripening while expression changes regarding its modification accompany berry softening

Cytoskeleton constituents and cell wall biosynthesis enzymes were over-represented among the annotated products of down-regulated transcripts (cluster FS7 in Fig. 4 and Table S8). Previous reports indicate no major changes in cell wall polysaccharide content during flesh [72] or skin ripening [73], which could be in line with the inactivation of cell wall biosynthesis without any noticeable activation of its degradation. Nevertheless, cellulose biosynthesis related expression seemed to be more intensely down-regulated in the flesh than in the skin (cluster S4 in Fig. 2 and Table S6) in view of the presence of six *CELLULOSE SYNTHASE* genes (*VIT_07s0005g02470*, *VIT_08s0007g03990*, *VIT_04s0069g00780*, *VIT_00s0414g00060*, *VIT_00s0975g00020*, and one unmatched probe set *VVTU4471_at*) more strongly repressed in the flesh (Table S5). The less pronounced inactivation of cellulose biosynthesis in the skin might respond to a faster cellulose turnover required in the more exposed external tissue, where cell wall is thicker [71] and contains more cellulose [72], [73].

‘Muscat Hamburg’ ripe berries are relatively soft [21] and the softening is gained mainly during veraison (Table 1). The highest loss of berry firmness was found between stages P and V1 with a loss of 83.8%, which corresponds to 97.9% of the total loss in firmness. Pectins are likely to be the key polymers for the mechanical strength of the cell wall. On this point, polygalacturonases (PG) are pectin-modifying enzymes that appear important in fruit softening [74], and several PG coding genes were identified as regulated with different expression profiles throughout ripening (Tables S5 and S7). In particular, the *VvPG1* transcript (*VIT_08s0007g08330*) displayed a tissue-differential accumulation pattern correlated with berry tissues softening, suggesting the involvement of this gene in the process, as shown for *FaPG1* in strawberry fruit softening [75], [76]. *VvPG1* was extremely up-regulated in flesh from veraison while its induction was considerably lower and only transient during veraison in the skin (Table S5). The increase in *VvPG1* expression after veraison has also been reported in ‘Shiraz’ berries [77] and its transient up-regulation in the skin has also been described in ‘Cabernet Sauvignon’ [78]. Conversely, other pectin modifying enzymes such as pectinesterases appear to be required in facilitating pectin breakdown [79]. However, the related transcripts did not show an accumulation pattern related to fruit softening. In this way, eight pectinesterase family transcripts (*VIT_07s0005g00730*, *VIT_07s0005g02440*, *VIT_11s0016g00290*, *VIT_11s0016g00300*, *VIT_11s0016g00330*, *VIT_05s0020g01110*, *VIT_04s0044g01010*, *VIT_16s0098g01900*) (cluster F3+4 from Fig. 2 and Table S5) showed an early down-regulation in flesh. Thus, the repression within this gene family does not support its role in berry softening and contrasts with the increased pectin-methylesterase activity observed throughout ripening [78].

Other cell wall modifying enzymes have also been involved in the loss of fruit firmness (reviewed in Prasanna *et al*. [79]) and several related transcripts differentially accumulated during ripening were detected. The transcriptomic analysis showed a significant enrichment of xyloglucan modifying enzymes among the transcripts similarly up-regulated in flesh and skin near maturity (cluster FS4 from Fig. 4 and Table S8). Six xyloglucan endotransglucosylase (XET) coding genes were included in cluster FS4 (Table S7; *VIT_11s0052g01300*, *VIT_11s0052g01280*, *VIT_11s0052g01270*, *VIT_11s0052g01260*, *VIT_11s0052g01200*, *VIT_11s0052g01180*). This is in the line of the findings reported by Pilati *et al*. [30], although here we show these expression changes to take place similarly in both flesh and skin. The role of XETs in fruit softening is not clear [74]. These genes were activated in the pericarp of already softened berries and they could participate in the last steps of berry expansion as XET have been involved in cell wall extensibility and growth [80]–[82]. Thus, the actual mechanisms working for grape softening, and the role of each of the cell wall modifying enzyme coding genes regulated throughout ripening, still needs further study to be determined and different mechanisms could be expected in table grapes selected for crispy texture and wine grapes intended to provide juicy musts.

#### A global view of gene expression does not clarify the mechanism of sugar accumulation in mesocarp cells

Phloem unloading in the berry becomes apoplastic around the onset of ripening [83]; therefore, plasmalemma transporters are required to import sugars into cells where they accumulate as hexoses inside the vacuole by means of vacuolar transporters [13]. Consistently with earlier reports in other cultivars [31], [84], [85], sugar accumulation in the ‘Muscat Hamburg’ berry from veraison (Table S2) was not accompanied by any particularly noticeable up-regulation of hexose transporter genes in the mesocarp. Only hexose transporter transcripts *VvHT2* (*VIT_18s0001g05570*) and *VvHT6* (*VIT_18s0122g00850*) were induced from veraison, and similarly in flesh and skin (clusters FS1 and FS2, respectively in Fig. 4 and Table S7). In contrast, the sugar transport functional category was over-represented among transcripts with higher accumulation in the skin from veraison (cluster S1+2 from Fig. 2 and Table S6). This profile included among other transcripts, *VvHT7* (*VIT_11s0149g00050*) and *VvGIN2* (*VIT_02s0154g00090*) encoding a vacuolar invertase presumably involved in sucrose hydrolysis previous to hexose transport into the vacuole. *VvGIN1* (*VIT_16s0022g00670*) also displayed higher expression in the skin than in the flesh, although it was down-regulated in both pericarp tissues (cluster S4 from Fig. 2 and Table S5). Altogether this could suggest that, in mesocarp cells, sugar accumulation throughout ripening is not directed by transcriptional changes at least regarding genes involved in mechanisms already described. Instead, higher sugar levels accumulated in mesocarp cells [10], [11] could feedback repress to a higher degree the expression of transcripts involved in sugar biosynthesis or transport [86].

The significant over-representation of the ‘starch biosynthesis’ functional category among genes up-regulated from V1 to ripeness in the pericarp (cluster FS1 in Fig. 4 and Table S8) was remarkable. The response was participated by the up-regulation of three transcripts encoding putative starch synthases (*VIT_10s0003g02880*, *VIT_16s0098g01780*, *VIT_10s0116g01730*), the enzyme involved in the addition of glucose residues to starch bonds. This is consistent with previous transcriptomic analysis in whole pericarp [31], [33] and, in addition, occurring in both pericarp tissues as reported in this analysis. However, the biological role of such expression changes is not evident since starch content decreases throughout berry ripening [31], [33], [87]. Induction of these genes within the pericarp could be triggered by high sugar levels [64], [88], [89], although the induction is not followed by starch accumulation probably because such accumulation in the berry could only result from *in situ* photosynthesis [87].

#### Transcripts encoding monoterpene biosynthetic enzymes not involved in Muscat aroma production are repressed throughout ripening in ‘Muscat Hamburg’

The Muscat aroma present in ‘Muscat Hamburg’ berries results from the accumulation of free monoterpenes [90]. Monoterpene content seems to similarly increase in flesh and skin throughout ripening [91] and its accumulation is dependent on cultivar genotype [92]. In ‘Muscat Hamburg’, using samples corresponding to the same stages as those analyzed in this research paper, Fenoll *et al*. [39] showed that most Muscat-related compounds (including linalool, nerol or citronellol) accumulated concomitantly with ripening.

Recent evidences indicate that gain-of-function mutations in the gene *VvDXS1* (*VIT_05s0020g02130*) encoding a 1-deoxy-D-xylulose-5-phosphate synthase, enzyme involved in the first steps of biosynthesis of terpenoid precursors, are the major determinants for terpenoid accumulation in Muscat varieties [93]–[96]. Interestingly, in the transcriptomic analyses carried out, this gene was down-regulated throughout ripening and to a higher extent in the flesh, while two other putative *DXS* (*VIT_18s0122g00610*, *VIT_00s0218g00110*) were similarly down-regulated in both tissues along pericarp ripening (cluster FS7 in Fig. 4 and Table S7). Thus, dominant mutations on *VvDXS1* should have direct effects on the enzymatic or regulatory properties of the DXS protein as has been recently demonstrated [96].

In this current study, the ‘monoterpene biosynthesis’ category was over-represented among those transcripts progressively down-regulated during ripening but to a lesser extent in the skin (cluster S3+4 from Fig. 2 and Table S6). Nonetheless, the related transcripts were not involved in the biosynthesis of the monoterpenes responsible of the Muscat flavor but in other geranylgeranyl-pyrophosphate-derived terpenoids. They included two genes annotated as isopiperitenol dehydrogenase (Table S5; *VIT_17s0000g05610*, *VIT_17s0000g05600*) and one putative neomenthol dehydrogenase (*VIT_15s0046g03570*). All three are in the menthol synthesis pathway not contributing to the Muscat flavor [97]. In addition, within the same cluster, a putative geranylgeranyl reductase (*VIT_17s0000g06280*) was identified, which consumes geranylgeranyl-pyrophosphate towards the production of porphyrins and terpenoid-quinone biosynthesis and three other genes involved in monoterpenoid-quinone biosynthesis (*VIT_15s0021g01040*, *VIT_05s0049g00400*, *VIT_13s0067g00110*). Thus, it is possible that the repression of other monoterpenoids biosynthesis could strengthen the accumulation throughout ripening of those participating in the Muscat aroma.

#### Response to pathogens is activated along the pericarp during ripening

Functional enrichment analyses evidenced the activation of pathogen defense gene expression responses in the pericarp upon berry ripeness (cluster FS4 in Fig. 4, Table S8). This enrichment is represented by the induction of several transcripts coding for pathogen-related (PR) proteins in both flesh and skin (Table S7). Concomitantly to PR genes up-regulation, PR proteins accumulation also increased throughout ‘Muscat Hamburg’ mesocarp ripening [61]. Deluc *et al*. [31] and Guillaumie *et* *al*. [34] also reported similar induction of *PR* genes in un-dissected ‘Cabernet Sauvignon’ and ‘Chardonnay’ ripening pericarps respectively, supporting it as a ripening developmental feature. *VvNPR1.1* (*VIT_11s0016g01990*), *VvNPR1.2* (*VIT_10s0042g01250*), and *PR1-like* genes (*VIT_03s0088g00710*, *VIT_03s0088g00700*) were among those found in the current study with this profile in ‘Muscat Hamburg’. *VvNPR1.1* and *VvNPR1.2* are orthologs of *AtNPR1*, a central regulator of the SA-mediated systemic acquired resistance [98]. Both genes are induced by SA and enhance *VvPR1* expression in grapevine leaves infected with *Plasmopara viticola* [99]. Thus the SA-mediated defense signaling pathway may operate to increase pathogen defenses once the berry is reaching maturity.

Phytoalexin biosynthesis coding transcripts were also up-regulated during berry ripening. This activation was evident after veraison and was significantly more intense in the skin despite affecting also the flesh (cluster S1 from Fig. 2 and Table S6). Twelve putative *STILBENE SYNTHASE* (*STS*) transcripts, responsible of resveratrol accumulation, presented this expression profile (Fig. 3). Pilati *et al*. [30] also observed up-regulated *STSs* during ‘Pinot Noir’ berry ripening although without taking into account the tissue bias, while increased expression of *STS* throughout ripening has also been reported in the skin of ‘Norton’ grapes [100]. Gatto *et al*. [101] reported increases in stilbene content during berry ripening in several grapevine cultivars concurrently with induction of *STS* and *PHENYLALANINE AMMONIA-LYASE* transcript expression. Similarly, in ‘Muscat Hamburg’ berries, two *PHENYLALANINE AMMONIA-LYASE* genes (*VIT_16s0039g01240*, *VIT_08s0040g01710*) showed the same expression profile as the cited *STSs* up-regulated preferably in the skin (cluster S1 from Fig. 2 and Table S5).

Interestingly, *WRKY* transcription factor coding transcripts (*WRKY6*-like: *VIT_10s0116g01200*, *WRKY18*-like: *VIT_04s0008g05760*, *WRKY27*-like: *VIT_02s0025g00420*, *WRKY39*-like: *VVTU14990_at*, *WRKY40*-like: *VIT_09s0018g00240*, *WRKY47*-like: *VIT_07s0005g02570*, *WRKY53*-like: *VIT_02s0025g01280* and *VIT_15s0046g01140*, *WRKY75*-like: *VIT_01s0010g03930*) were up-regulated with the same profile as the STS transcripts and also with higher intensity in the skin. Additionally, ultraviolet-B radiation in ‘Malbec’ leaves [40] and powdery mildew infection on those of ‘Cabernet Sauvignon’ [102], also provoked a correlated induction of *WRKY* and *STS* transcripts, a concert being suggestive of a direct regulation. Indeed, VvWRKY1 is able to activate the promoter of elicitor-responsive genes, which are induced together to *STSs* in grapevine plantlets [103], although *VvWRKY1* was among the transcripts similarly induced in skin and flesh after pre-veraison (*VIT_17s0000g01280*; cluster FS1 in Fig. 4, and Table S7). Further studies would be required to test the specific role of these WRKY TFs in the regulation of *STS* genes during berry ripening.

Globally, activation of pathogen defenses in the berry pericarp near maturity appears as a characteristic feature of grape ripening given the healthy aspect of the fruit and the consistency of the two years analyzed. In fact, accumulation of antifungal proteins concurrently to sugars during pericarp ripening has previously been reported [104]. This response might have been preventively triggered during the ripening developmental program to protect the berry once it is becoming softer and rich in sugars (Table S2) and, therefore, more suitable for biotrophic pathogen attacks.

#### Expression of auxin signaling decays along the pericarp upon veraison

Transcriptional profiling indicates a down-regulation of genes involved in auxin signaling after pre-veraison in both pericarp tissues (Tables S4, S6, and S8). This was the case of several *AUXIN RESPONSE FACTOR* (*ARF*) genes (*ARF1*-like: *VIT_18s0089g00910*; *ARF2*-like: *VIT_11s0065g00310*; *ARF3*-like: *VIT_10s0003g00420*; *ARF4*-like: *VIT_06s0004g03130*; *ARF6*-like: *VIT_00s1203g00010*; *ARF10*-like: *VIT_13s0019g04380*; *ARF18*-like: *VIT_15s0046g00290*). ARF proteins regulate expression of auxin-response genes but these need interaction with Aux/IAA (auxin/indole acetic acid) proteins to be sensitive to auxin levels [105]. Aux/IAA repressors heterodimerize with ARFs resulting in the repression of auxin-response genes [106]. Four *Aux/IAA* (auxin/indole acetic acid) transcripts (*IAA9*-like1: *GSVIVT00013515001*; *IAA9*-like2: *VIT_18s0001g08090*; *IAA9-*like3: *VIT_09s0002g04080*; *IAA12/BODENLOS*-like: *VIT_07s0005g04380*) and a *TIR1* transcript (*VIT_07s0104g01320*), homologous to the Arabidopsis auxin receptor [107], were down-regulated in flesh and skin with the same pattern as the *ARF*-like transcripts. Altogether these results suggest a general decay in auxin response and responsiveness in the pericarp from veraison to ripeness. These results are in agreement with previous reports indicating that the auxin levels decline before the onset of ripening [108]–[110] as well as with the inhibitory effects of this hormone on different aspects of berry ripening [62], [111], [112]. Therefore attenuation of auxin signaling might be important to turn off green berry-related processes allowing for those involved in ripening to be activated.

In addition to the shared flesh and skin down-regulation of common auxin signaling components, some auxin-related transcripts also showed significant tissue-specific regulation (Tables S5 and S6). These tissue divergences might be due to the regulation of tissue-specific auxin-responses and/or to the requirement of tissue-specific elements for the auxin signal transduction pathway. Directed analysis would be required to determine the implication of each of these regulators in grape berry development.

#### Gibberellin biosynthetic genes were inactivated in the pericarp from veraison

‘Diterpenoid metabolism’ was a functional category over-represented among the cluster of transcripts down-regulated from veraison in both exocarp and mesocarp (cluster FS5 in Fig. 4 and Table S8). It is worth mentioning within the cited functional category the presence of a transcript encoding an *ent*-kaurenoic acid oxidase (*VIT_18s0001g10500*) and three encoding gibberellin (GA) 20-oxidases (*VIT_18s0001g06690*, *VIT_04s0044g02010*, *VIT_19s0140g00140*). The former enzyme catalyzes one of the early steps in GA biosynthesis [113] and the later the limiting biosynthetic step for the accumulation of bioactive GA [114]. Therefore, these expression results are consistent with a reduction in the production of bioactive GA along the pericarp from the onset of ripening [108] and suggest that this phytohormone is more likely involved in the regulation of pre-veraison pericarp developmental events.

### Conclusions

A transcriptional analysis of grape berry ripening was carried out considering separately processes taking place in flesh and skin tissues. In addition, an in depth functional categorization of probesets on the GrapeGen GeneChip® utilized has provided a complementary tool to interpret the functional meaning of transcriptional changes throughout ripening and of the differences between both pericarp tissues. The results of the PCA analysis as well as the directed comparisons indicated that a large amount of the transcriptional program operating during ripening is shared between grape exocarp and mesocarp but also highlight important tissue differences. An apparent deactivation of green stage-related processes dominates the common pericarp program, which seems to take place more rapidly in the flesh than in the skin. Furthermore, skin ripening can be considered a more elaborated process in view of the higher number of functional categories active during exocarp ripening. This higher complexity, at least in terms of gene expression, is supported not only by the identification of more skin-specific activated processes such as stilbenoid biosynthesis or macromolecule transport, but also by the higher expression and maintenance in skin than in flesh of transcripts related to other processes like anthocyanin biosynthesis. This analysis also provides a number of candidate genes to account for the regulation of tissue-specific ripening events. Altogether, these findings provide the basis to understand the ripening process in the berry pericarp and its regulation. They constitute a starting point for further characterization of this relevant grape developmental process and help to focus breeding programs aimed at improving grape quality traits determined by specific berry tissues.

## Materials and Methods

### Sample Collection

Samples from *Vitis vinifera* cv. ‘Muscat Hamburg’ were collected along berry ripening from the Torreblanca Vineyard (experimental vineyard at the Instituto Murciano de Investigación y Desarrollo Agrario y Alimentario located in Torreblanca, Murcia, Spain) during the 2005 and 2006 summer seasons. Berries were weekly picked from bunches (one bunch per vine from each selected vine) from June to August each year. For each sampling and vine, berries were classified in five ripening stages that were estimated using different non-destructive methods. Pre-veraison samples were harvested as green hard berries of at least 15 mm in size according to touch-assessed hardiness. Veraison berries with half or full coloured skin surface corresponded to 50% and 100% veraison samples. After veraison ripening berries were classified according to their floatability in solutions with different NaCl densities, an indication of the internal sugar concentration [115]. At this stage berries were sampled with densities between 110–130 g NaCl/L for R1 and between 130–150 g NaCl/L for R2, which corresponded to commercially ripen table grape berries.

For most fruit measurements thirty berries were considered per biological replica. Equatorial diameter was measured with a Mitutoyo Digimatic caliper (Japan). Dry weight percentage was determined by measuring the difference between fresh and dry weight after drying in an oven at 60°C. For firmness determinations berries from R1 and R2 stage were subjected to a deformability test with a texture analyzer (TA.XT plus, Texture Technologies Corp., Scarsdale, N.Y., U.S.A./Stable Micro Systems, Godalming, Surrey, U.K.) equipped with a stainless steel plate and a 100-mm stainless steel probe. Settings were pre-test speed of 10 mm sec^−1^, test speed of 2 mm sec^−1^ and post-test speed of 10 mm sec^−1^. Firmness was measured as the force (Newton) needed to deform the berry by 20% at its equatorial diameter and expressed as loss of firmness with respect to the initial firmness (stage P).

For anthocyanin and phenolic compounds determinations homogeneous samples of approximately 100 berries from each biological replica were separated into skins and pulps. Phenolic compounds were extracted and determined following a slight modification of the method described by Cantos *et al*. [116]. Skin samples of three grams were homogenized in an extraction solution containing methanol/formic acid, (97∶3 v/v) using a Politron (PT-MR 3100, Switzerland). The extracts were centrifuged at 10000 g and the remaining pellet was re-extracted using fresh extraction solvent, vortexed for one minute and centrifuged. This procedure was repeated twice. Finally, the combined extracts were evaporated to dryness under vacuum at <35°C, and the residue was dissolved in two mL of extraction solution. All samples were passed through 0.45 µm filters prior to spectrophotometric techniques. Triplicate extractions were prepared from each biological replica of the ripening stages. All extractions and analyses were carried out in the dark to protect the phenolic compounds from degradation. Total phenolic content in the skin extract was determined spectrophotometrically using the Folin-Ciocalteu method [117] adapted to a microplate. The absorbance of the blue colour produced was measured with a spectrophotometer Shimadzu at 750 nm (UV-2401PC, Japan) using gallic acid as standard. Total anthocyanins were estimated spectrophotometrically at 540 nm [118] using malvidin 3-glucoside (Extrasynthese, France) as standard.

For specific sugars and organic acids determinations, whole grape samples of approximately 50 berries for each biological replica were homogenized in Politron (PT-MR 3100, Switzerland) with 50 mL of ultrapure water at 50°C. The extracts were centrifuged and the supernatant was recovered, filtered and purified by C_18_ SEP PAK cartridges. For sugars, samples were analyzed using an HPLC system (Hewlett-Packard, Germany) equipped with a refraction index detector. Separation was performed on a 305×7.8 mm i.d., 9 µm ionic-exchange HC-75 column (Transgenomic, Ltd., Cheshire, UK) with ultra-pure water as the mobile phase. Organic acids were analyzed using a photodiode array UV/Vis detector. The separation was done on a 250×4.6 mm i.d., 5 µm Mediterranean Sea 18 (Teknokroma, Barcelona, Spain). Readings were carried at 210 nm.

### Affymetrix GrapeGen GeneChip®

A custom Affymetrix GeneChip® was developed as a result of the Spanish-Canadian GRAPEGEN project [119]. GeneChip® information is deposited in ArrayExpress [40] and GEO [100] under accession numbers E-MEXP-2541 and GSE24561, respectively. The GeneChip® contains 23,096 probe sets corresponding to 18,711 non redundant transcripts (about 66% increment with respect to the previous Affymetrix commercially available chips with 11,149 non redundant probe sets [120]). Each probe set comprised eleven 25-mer probes. The probe sets represent *Vitis vinifera* EST sequences from consensus contigs (when overlapped) mostly from ‘Cabernet Sauvignon’, ‘Muscat Hamburg’, ‘Pinot Noir’, ‘Chardonnay’ and ‘Shiraz’ cultivars. Sequences used for probe design were obtained from public databases and from our own EST sequencing project [119]. All probe sets were manually re-annotated and functionally categorized in order to perform functional enrichment analyses. Additionally, a web tool, GrapeGenDB (http://bioinfogp.cnb.csic.es/tools/GrapeGendb/), was developed for accessing updated annotations on the probe sets of the GrapeGen Affymetrix custom array (GrapeGena520510F). With the web tool, users can define a subset of interesting probe sets and select the information fields of interest. Accession codes to CRIBI 12×v1 or in its defect Genoscope vitis 8× public databases are automatically linked in the results page. The consensus sequence of each probe set is shown together with several one-click links to standard homology searching programs. These searches can be easily carried-out against nucleotide (blastn) or translated protein (blastx) [121] sequence databases either general or Vitis-specific. Also, a link to BLAT program to search homologies against the Genoscope Vitis sequence is included. Finally, users can download the results table in Excel format for later analysis.

### Annotation of GrapeGen GeneChip® Features

The probeset sequences were matched with the 12× genome with Megablast using the following criteria for positive matching: homology higher than 95% on a length larger than 100 bp and an e-value <e−20. Annotations performed for the 8× genome in Grimplet *et* *al*. [120], updated for the 12×v1 was inferred to the corresponding probesets [41]. Probesets not matching a gene were newly annotated according to the method described in Grimplet *et* *al*. [120]. Functional categorization was performed by manually attributing function to the probesets according to a custom-made catalog based on plant-related terms from the GO terms and MIPS FunCat [122], [123]. A hierarchical classification was carried out, starting from 12 higher level terms and reaching up to eight descendant levels. GrapeGen GeneChip® probesets were included in a total of 516 categories, which can be consulted in Table S3 and GrapeGenDB.

### RNA Isolation and GeneChip® Hybridization

Total RNA was extracted from berry tissues according the procedures described by Reid *et al*. [124]. DNase digestion of contaminating DNA in the RNA samples was carried out with the RNase-Free DNase Set (QIAGEN). Final RNA purification was performed using the RNeasy Mini Kit (QIAGEN) according to standard protocols. Samples were hybridized at the Genomics Unit at the National Biotechnology Centre (CNB-CSIC, Madrid). RNA integrity analysis was performed with an Agilent́s Bioanalyzer 2100. RNA labelling, arrays hybridization, washing, staining, and scanning with the GeneChip® Scanner 3000 were performed as indicated in PlexDB (VV33).

### Microarray Data Analysis

#### Preprocessing and PCA

The full GeneChip® raw expression dataset is available in PlexDB under the accession number VV33. Signal values from all the microarray hybridizations were normalized together using Robust Microarray Average (RMA) [125] by RMAExpress. After the average of expression values for redundant probesets using Babelomics preprocessing tools [126], a PCA [127] was directed over the normalized dataset. The six first PCs were analyzed using Acuity 4.0 (Axon Molecular Devices, http://www.moleculardevices.com). Gene PCA LS were obtained for the first three PCs (Table S3).

#### Identification of differentially accumulated transcripts and clustering

To detect differential expression between the developmental series of flesh and skin samples, a two-class paired time course SAM [128], a suitable method to pick out significant genes based on differential expression between temporal series, was conducted with SAM package (http://www-stat.stanford.edu/~tibs/SAM/). A 1% FDR was applied in the SAM analysis considering either the slope or the signed area for the time course summary to identify probesets either with constant or sharp transient changes respectively. Cut-off of 3-fold difference between flesh and skin in one developmental stage, or 2-fold difference in two developmental stages, was applied (Table S5). On the other hand, flesh and skin were considered the same kind of sample, in a five-class one-way Analysis of Variance (ANOVA) [129] applied in MeV [130], in order to find transcripts changing during berry development with a similar pattern in both tissues. ANOVA was performed on probesets that did not result differentially expressed between flesh and skin. Significant probesets (Table S7) were considered according to a *p*-value <0.01, after Bonferroni adjusted correction for multiple testing in the ANOVA, and with at least 3-fold difference between any pair of stages, or at least 2-fold difference in two pairs of stages. In this analysis, a more conservative *p*-value correction was applied to counteract the statistic stability provided by the availability of 12 replicas per group after joining the two tissues. The significant transcripts identified from each analysis were grouped according to shared developmental expression patterns. Self-organizing maps (SOM) [131] with Euclidean squared and Scale rows metrics was applied in Acuity 4.0 for the clustering. Eight groups were generated in a 4×2 clustering in both cases as estimated by gap statistics [132] in Acuity 4.0.

#### Functional analysis

Selected gene lists were further analyzed with FatiGO [45] to identify significant enrichment of ‘GrapeGen custom functional annotation’ terms. The lists analyzed resulted from the SOM groups and their combinations, and also from the genes showing higher contribution to the two first PCs of the PCA (|PCA LS| >10). Genes negatively or positively determining each PC were analyzed separately. Probesets from each list were compressed to non-redundant transcripts prior to the functional analysis. This was done by averaging the expression values using Babelomics preprocessing tools. Fisher’s exact test was carried out in FatiGO to compare each study list to the list of total non-redundant transcripts housed in the GrapeGen GeneChip. Significant enrichment was considered in case of *p*-value <0.05 after Benjamini and Hochberg correction for multiple testing. Expression values of the differentially accumulated transcripts were uploaded into VitisNet updated to the 12×v1 gene predictions [41] with the cytoscape software [133]. Networks for the phenylpropanoids, flavonoids, and anthocyanin biosynthesis were merged into a single network (Fig. 3), branches containing only non-differentially accumulated transcripts were trimmed out.

## Supporting Information

Figure S1 **PCA plot of ‘Muscat Hamburg’ pericarp samples according to their expression data.** **A**, PCA plot of flesh and skin ripening samples according to their RMA expression data. The first (PC1) and the third (PC3) principal components are represented (from a six component PCA after data centering). Expression data from probe sets matching the same transcript were averaged before the PCA. Six samples corresponding to three biological replicas collected in two different years were analyzed per developmental stage. Green, pre-veraison (>15 mm); yellow, 50% veraison; red, 100% veraison; blue, ripe 1; purple, ripe 2. Flesh: light color; Skin: dark color. **B**, PC3 stage averaged loading scores for flesh and skin. Blue, flesh; dark red, skin. Error lines indicate stage replicates standard deviation.
(TIF)

Figure S2 **PCA plot of the unique transcripts according to their expression in flesh and skin throughout ripening.** **A**, PC1 and PC2 are represented. Transcripts most contributing to the distribution in the PC1 (|PC1 loading score| >10) are highlighted. **B**, Expression profile of transcripts with |PC1 loading score| >10. **C**, PC1 and PC2 are represented. Transcripts most contributing to the distribution in the PC2 (|PC2 loading score| >10) are highlighted. **D**, Expression profile of transcripts with |PC2 loading score| >10. **E**, PC1 and PC3 are represented. Transcripts most contributing to the distribution in the PC3 (|PC3 loading score| >10) are highlighted. **F**, Expression profile of transcripts with |PC3 loading score| >10. P, pre-veraison (>15 mm); V1, 50% veraison; V2, 100% veraison; R1, ripe 1; R2, ripe 2. Purple, transcripts positively determining the PC1 (PC1 score >10); green, transcripts negatively determining the PC1 (PC1 score <−10); yellow, transcripts positively determining the PC2 (PC2 score >10); red, transcripts negatively determining the PC2 (PC2 score <−10); blue, transcripts positively determining the PC3 (PC3 score >10); orange, transcripts negatively determining the PC3 (PC3 score <−10).
(TIF)

Table S1 Additional physiological data at different stages of berry ripening.
(DOCX)

Table S2 Ripening evolution of individual soluble sugars and organic acids.
(DOCX)

Table S3 Unique transcript PCA loading scores, annotation, and functional categorization.
(XLSX)

Table S4 Functional categories enriched in the unique transcripts with higher PCA loading scores.
(XLSX)

Table S5 Probesets showing significant differences between flesh and skin throughout berry ripening.
(XLSX)

Table S6 Functional enrichment in clusters of transcripts differentially accumulated between flesh and skin.
(XLSX)

Table S7 Probesets showing significant changes throughout ripening and taking place in the same way in flesh and skin.
(XLSX)

Table S8 Functional enrichment in clusters of transcripts changing in the same way in flesh and skin throughout ripening.
(XLSX)
