# Correction: Berry Flesh and Skin Ripening Features in *Vitis vinifera* as Assessed by Transcriptional Profiling

**DOI:** 10.1371/annotation/fd93800a-3b3c-484d-97a9-190043309e4b

**Published:** 2012-10-10

**Authors:** Diego Lijavetzky, Pablo Carbonell-Bejerano, Jérôme Grimplet, Gema Bravo, Pilar Flores, José Fenoll, Pilar Hellín, Juan Carlos Oliveros, José M. Martínez-Zapater

The following reference was erroneously omitted from the published manuscript:

Hemert J, Hong L, Wise RP, Dickerson JA. PLEXdb: gene expression resources for plants and plant pathogens. Nucleic Acids Res. 40(D1):D1194-D1201) 

