# Supplementary figures and images for: Berry Flesh and Skin Ripening Features in Vitis vinifera as Assessed by Transcriptional Profiling
Source: PLoS One. 2012 Jun 29;7(6):e39547. doi: 10.1371/journal.pone.0039547 (PMC3386993; doi:10.1371/journal.pone.0039547)

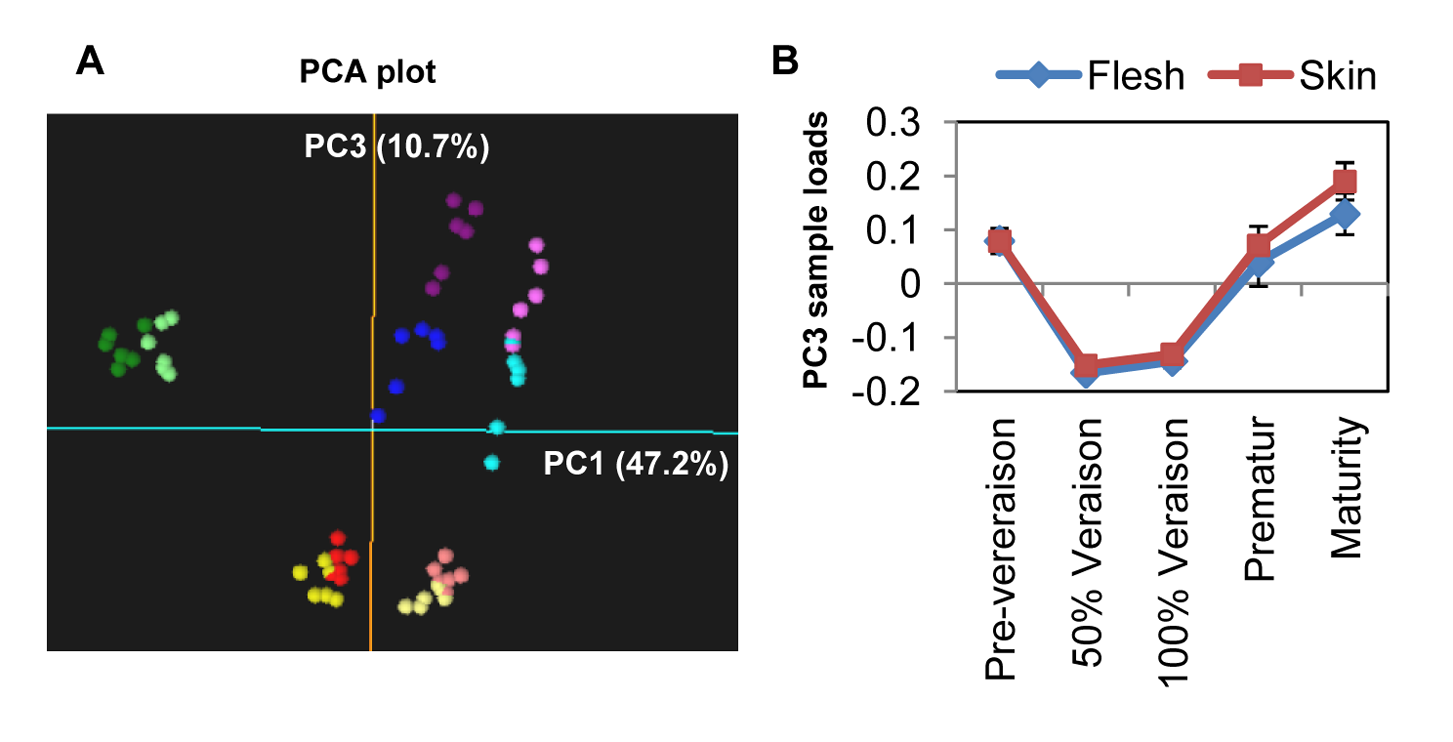

Supplement: Figure S1 — PCA plot of ‘Muscat Hamburg’ pericarp samples according to their expression data. A, PCA plot of flesh and skin ripening samples according to their RMA expression data. The first (PC1) and the third (PC3) principal components are represented (from a six component PCA after data centering). Expression data from probe sets matching the same transcript were averaged before the PCA. Six samples corresponding to three biological replicas collected in two different years were analyzed per developmental stage. Green, pre-veraison (>15 mm); yellow, 50% veraison; red, 100% veraison; blue, ripe 1; purple, ripe 2. Flesh: light color; Skin: dark color. B, PC3 stage averaged loading scores for flesh and skin. Blue, flesh; dark red, skin. Error lines indicate stage replicates standard deviation. (TIF) [file pone.0039547.s001.tif]

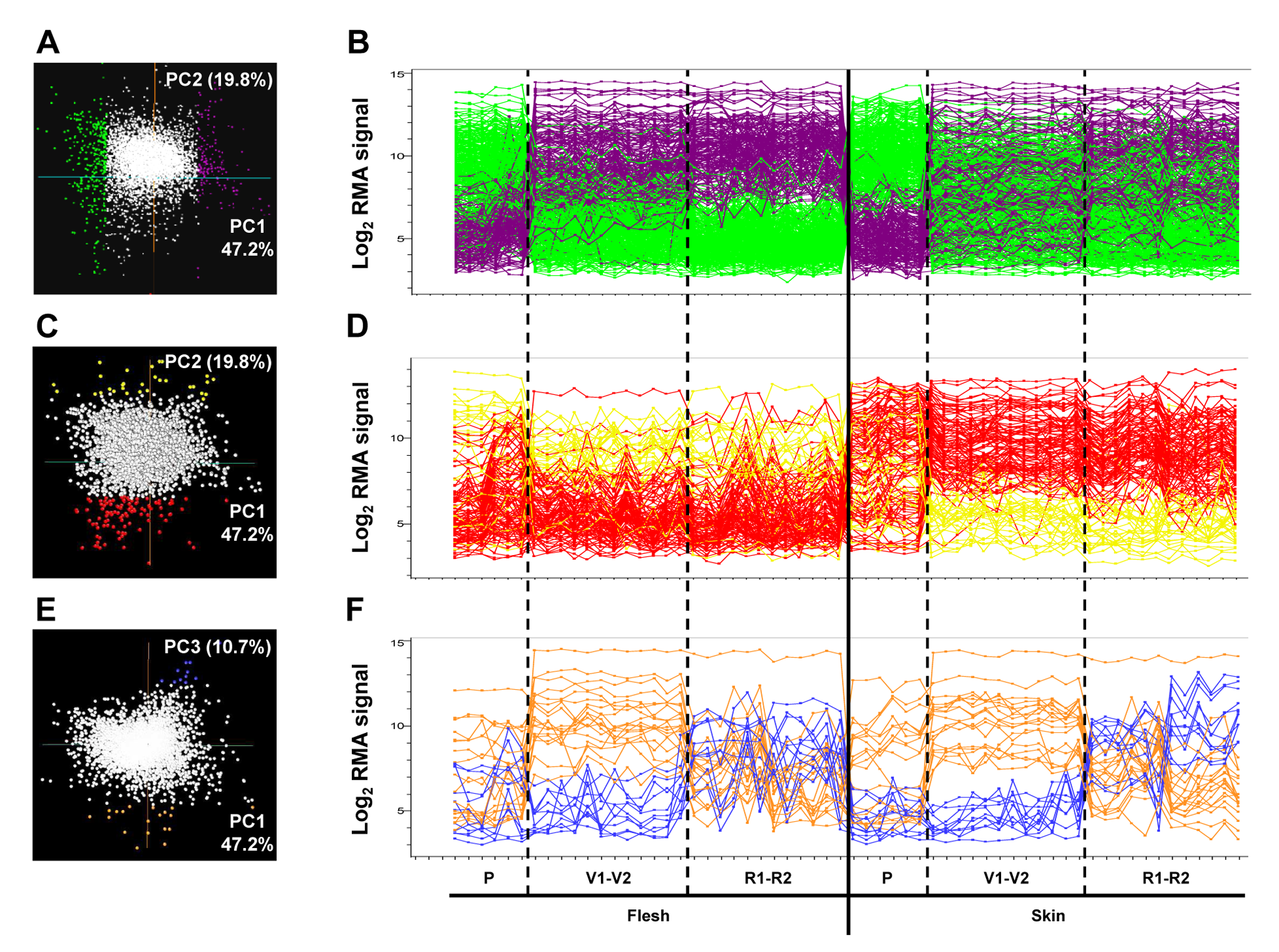

Supplement: Figure S2 — PCA plot of the unique transcripts according to their expression in flesh and skin throughout ripening. A, PC1 and PC2 are represented. Transcripts most contributing to the distribution in the PC1 (|PC1 loading score| >10) are highlighted. B, Expression profile of transcripts with |PC1 loading score| >10. C, PC1 and PC2 are represented. Transcripts most contributing to the distribution in the PC2 (|PC2 loading score| >10) are highlighted. D, Expression profile of transcripts with |PC2 loading score| >10. E, PC1 and PC3 are represented. Transcripts most contributing to the distribution in the PC3 (|PC3 loading score| >10) are highlighted. F, Expression profile of transcripts with |PC3 loading score| >10. P, pre-veraison (>15 mm); V1, 50% veraison; V2, 100% veraison; R1, ripe 1; R2, ripe 2. Purple, transcripts positively determining the PC1 (PC1 score >10); green, transcripts negatively determining the PC1 (PC1 score <−10); yellow, transcripts positively determining the PC2 (PC2 score >10); red, transcripts negatively determining the PC2 (PC2 score <−10); blue, transcripts positively determining the PC3 (PC3 score >10); orange, transcripts negatively determining the PC3 (PC3 score <−10). (TIF) [file pone.0039547.s002.tif]
