# Supplementary material for: Berry Flesh and Skin Ripening Features in Vitis vinifera as Assessed by Transcriptional Profiling
Source: PLoS One. 2012 Jun 29;7(6):e39547. doi: 10.1371/journal.pone.0039547 (PMC3386993; doi:10.1371/journal.pone.0039547)
Supplement: Table S1 — Additional physiological data at different stages of berry ripening. (DOCX) [file pone.0039547.s003.docx]

| **Table S1.** Additional physiological data at different stages of berry ripening. | | | | | |
| --- | --- | --- | --- | --- | --- |
| Parameter (year) | P | V1 | V2 | R1 | R2 |
| SSC (2005) | - | 13,2±0,00 | 15,1±0,07 | 17,7±0,07 | 19,7±0,07 |
| SSC (2006) | 4,6±0,07 | 13,2±0,17 | 14,9±0,03 | 16,8±0,06 | 19,4±0,40 |
| Total acidity (2005) | - | 17,7±0,78 | 15,6±0,45 | 10,0±0,68 | 6,2±0,05 |
| Total acidity (2006) | 31,7±0,83 | 17,2±0,32 | 14,2±0,24 | 9,2±0,20 | 5,2±0,08 |
| pH (2005) | - | 2,8±0,01 | 3,0±0,00 | 3,2±0,01 | 3,4±0,01 |
| pH (2006) | 2,7±0,00 | 2,9±0,02 | 2,9±0,01 | 3,3±0,01 | 3,5±0,03 |
| CIRG (2005) | - | 2,3±0,14 | 4,3±0,08 | 5,2±0,08 | 5,5±0,04 |
| CIRG (2006) | 0,8±0,01 | 2,6±0,15 | 3,9±0,11 | 5,2±0,08 | 5,5±0,12 |
| This data were previously published by Fenoll et al (2009) and correspond to the same samples used in the present work. Total soluble solids content (SSC, ºBrix), total acidity (g tartaric·L^-1^), pH, and colour (CIRG) throughout ripening of Muscat of Hamburg during 2005 and 2006. P, pre-véraison (15 mm); V1, 50% véraison; V2, 100% véraison; R1, 110-130 g NaCl·L^-1^; R2, 130-150 g NaCl·L^-1^ | | | | | |
