# Supplementary material for: Berry Flesh and Skin Ripening Features in Vitis vinifera as Assessed by Transcriptional Profiling
Source: PLoS One. 2012 Jun 29;7(6):e39547. doi: 10.1371/journal.pone.0039547 (PMC3386993; doi:10.1371/journal.pone.0039547)
Supplement: Table S2 — Ripening evolution of individual soluble sugars and organic acids. (DOCX) [file pone.0039547.s004.docx]

| **Table S2.** Evolution of individual soluble sugars and organic acids. | | | | | | |
| --- | --- | --- | --- | --- | --- | --- |
| **Compound (year)** | **P** | **V1** | **V2** | **R1** | **R2** |  |
| **Glucose (2005)** | 190.1 ± 0.2 | 234.6 ± 1.3 | 273.9 ± 0.7 | 292.0 ± 2.5 | 262.6 ± 5.4 |  |
| **Glucose (2006)** | 207.7 ± 0.2 | 272.7 ± 3.1 | 299.4 ± 2.8 | 331.5 ± 4 | 343.1 ± 1.2 |  |
| **Fructose (2005)** | 16.9 ± 0 | 237.0 ± 1.7 | 294.9 ± 1 | 330.3 ± 3 | 313.1 ± 3.5 |  |
| **Fructose (2006)** | 24.6 ± 0.1 | 276.6 ± 3.4 | 320.1 ± 3.5 | 361.2 ± 4.4 | 391.7 ± 1.4 |  |
| **Sucrose (2005)** | N/d | 1.2 ± 0 | 7.4 ± 0.3 | 7.4 ± 0.1 | 9.8 ± 1.1 |  |
| **Sucrose (2006)** | 1.5 ± 0 | 3.2 ± 0 | 4.3 ± 0.1 | 4.5 ± 0.2 | 7.3 ± 0.2 |  |
| **Tartaric acid (2005)** | 107.0 ± 0.1 | 32.7 ± 0.1 | 19.3 ± 0 | 19.1 ± 0.2 | 14.5 ± 0.1 |  |
| **Tartaric acid (2006)** | 120.0 ± 0.1 | 24.0 ± 0.3 | 22.6 ± 0.1 | 17.4 ± 0.2 | 11.0 ± 0.1 |  |
| **Malic acid (2005)** | 271.8 ± 0.1 | 65.4 ± 0.1 | 24.4 ± 0.2 | 25.5 ± 0.1 | 13.6 ± 0.2 |  |
| **Malic acid (2006)** | 315.4 ± 0.4 | 55.2 ± 0.6 | 31.1 ± 0.1 | 21.3 ± 0.3 | 11.5 ± 0 |  |
| **Citric acid (2005)** | 1.97 ± 0.01 | 0.49 ± 0.01 | 0.45 ± 0 | 0.48 ± 0.1 | 0.37 ± 0.1 |  |
| **Citric acid (2006)** | 1.85 ± 0.01 | 0.45 ± 0.01 | 0.49 ± 0.1 | 0.45 ± 0.1 | 0.32 ± 0.1 |  |
| All measures are shown as mg·g^-1^ dry weight grape. P, pre-véraison; V1, 50% véraison; V2, 100% véraison; R1, 110-130 g NaCl·L^-1^; R2, 130-150 g NaCl·L^-1^. Data are ± SD (n=3). | | | | | |  |
